# Sociodemographic Factors and Treatment Patterns Associated with Overall Survival in Splenic Marginal Zone Lymphoma: A Nationwide Retrospective Cohort Study (2000–2022)

**DOI:** 10.3390/cancers18081300

**Published:** 2026-04-20

**Authors:** Manas Pustake, Oboseh Ogedegbe, Atulya Aman Khosla, Sakditad Saowapa, Mohammad Arfat Ganiyani, Avi Harisingani, Nishant Tiwari, Stevenson Ongsyping, Jesus Gomez

**Affiliations:** 1 Department of Internal Medicine, Texas Tech University Health Sciences Center El Paso, El Paso, TX 79905, USA or pustakemanas@gmail.com (M.P.); stevenson.ongsyping@ttuhsc.edu (S.O.); 2 Department of Internal Medicine, Trinity Health Ann Arbor Hospital, Ypsilanti, MI 48197, USA; 3 Department of Internal Medicine, Corewell Health William Beaumont University Hospital, Royal Oak, MI 48073, USA; 4 Department of Hematology and Oncology, University of Iowa Health Care Medical Center, Iowa City, IA 52242, USA; 5 Department of Medical Oncology, Miami Cancer Institute, Baptist Health South Florida, Miami, FL 33176, USA; mohammadarfat.ganiyani@baptisthealth.net; 6 Department of Internal Medicine, Loyola MacNeal Hospital, Berwyn, IL 60402, USA; avih1997@gmail.com; 7 Department of Hematology and Oncology, Stephenson Cancer Center, University of Oklahoma Health, Oklahoma City, OK 73104, USA; dr.nishant.tiwari.1996@gmail.com; 8 Department of Hematology and Oncology, University Medical Center of El Paso, El Paso, TX 79905, USA

**Keywords:** splenic marginal zone lymphoma, SEER, overall survival, Cox proportional hazards, sociodemographic factors, marital status, Hispanic ethnicity, systemic antineoplastic therapy, population-based study, competing risks

## Abstract

**Background**: Splenic marginal zone lymphoma (SMZL) is a rare indolent lymphoma with extremely limited population-level evidence on social and treatment correlates of survival. **Methods**: We conducted a retrospective cohort study using SEER (2000 to 2022) to evaluate OS in primary SMZL (ICD O 3 9689; spleen C42.2). We summarized baseline features and treatments and used Kaplan–Meier and Cox regression. **Results**: The cohort included 3548 patients (mean age: 68.2 years; 53.6% female). Most were White (89.8%) and non-Hispanic (92.1%). The Ann Arbor stage was missing in 39.4%. Treatment included systemic antineoplastic therapy in 26.4%, beam radiation in 0.7%, and primary site surgery in 21.4%. At last follow-up, 56.8% were alive; non-Hodgkin lymphoma accounted for 15.8% deaths in the cohort, with substantial competing causes including heart disease (6.1%). In multivariable Cox analysis, OS was independently associated with age (HR 1.082 per year, 95% CI 1.072–1.091), male sex (HR 1.34, 95% CI 1.14–1.57), Hispanic ethnicity (HR 1.42, 95% CI 1.08–1.88), systemic antineoplastic therapy (HR 1.42, 95% CI 1.18–1.70), divorced/separated marital status vs. married (HR 1.35, 95% CI 1.03–1.77), and stage IV disease (HR 1.70, 95% CI 1.16–2.50). Race and year of diagnosis were not independently associated with OS in the adjusted model. **Conclusions**: In our large population-based analysis, OS in SMZL tracks with demographic and social variables and competing risks. Stage missingness and treatment selection limit causal inference for management effects.

## Simple Summary

Splenic marginal zone lymphoma is a rare blood cancer that mainly affects older adults. Because it is uncommon, doctors rely on limited data to understand which factors truly influence survival. We studied 3548 patients in a large national cancer registry from 2000 to 2022 to identify which patient characteristics and treatments were linked to overall survival. We found that age, sex, ethnicity, and marital status were independently associated with survival. Older age, male sex, and Hispanic ethnicity were linked to a higher risk of death. Married patients had better survival compared with divorced or separated individuals. Systemic antineoplastic therapy was associated with was associated with increased risk of death, likely reflecting that sicker patients were more likely to receive it. Surgery and radiation were not associated with longer survival. Many deaths were due to causes other than lymphoma, such as heart and lung disease. These findings highlight the importance of comprehensive medical and supportive care alongside cancer treatment.

## 1. Introduction

Splenic marginal zone lymphoma (SMZL) is a rare, indolent B-cell non-Hodgkin lymphoma characterized by primary involvement of the spleen, bone marrow, and peripheral blood, typically presenting in older adults with splenomegaly and cytopenias [1,2,3]. Accounting for less than 2% of all lymphoid malignancies, SMZL exhibits a highly heterogeneous clinical course, ranging from prolonged survival with minimal intervention to progressive disease requiring systemic therapy [4,5]. Despite its generally favorable prognosis compared with aggressive lymphomas, long-term outcomes are influenced by a complex interplay of patient characteristics, disease biology, and treatment selection [4,5,6,7].

Management of SMZL has evolved substantially over the past two decades. Historically, splenectomy played a central role in both diagnosis and symptom control, often producing durable hematologic responses [4,5]. More recently, the introduction of immunochemotherapy and anti-CD20-based regimens has shifted practice patterns toward non-surgical management, particularly for patients with advanced age, comorbidities, or disseminated disease [5,8]. However, owing to the rarity of SMZL, prospective randomized trials are limited, and clinical decision-making frequently relies on retrospective studies, small series, and expert consensus rather than high-level evidence [4,5,8].

In addition to disease- and treatment-related factors, sociodemographic characteristics such as sex, race, ethnicity, marital status, and socioeconomic context have emerged as important determinants of survival across multiple hematologic malignancies [9]. These factors may reflect differences in baseline health, comorbidity burden, access to care, treatment tolerance, social support, and competing risks of mortality, particularly in indolent lymphomas affecting older populations [9,10,11,12]. However, the extent to which sociodemographic factors independently influence survival in SMZL remains poorly characterized, with prior studies limited by small sample sizes, short follow-up, or incomplete adjustment for competing causes of death [7,13,14].

To address these gaps by leveraging large-scale analyses of rare malignancies with extended follow-up, we performed this study using the Surveillance, Epidemiology, and End Results (SEER) dataset. This dataset captures detailed demographic, diagnostic, treatment, and survival information across diverse U.S. populations, making it a valuable resource for evaluating real-world outcomes in rare malignancies like SMZL [15]. Importantly, in indolent lymphomas such as SMZL, where non-lymphoma mortality is substantial and cause-of-death attribution may be unreliable, overall survival represents a particularly robust and clinically meaningful endpoint for registry-based research [16].

We hypothesized that sociodemographic factors and treatment patterns are independently associated with overall survival in SMZL, reflecting both disease-related risk and competing non-lymphoma mortality. Compared with prior SEER-based studies, our analysis uses a larger and more contemporary cohort with extended follow-up through 2022 and incorporates a more comprehensive evaluation of sociodemographic variables alongside treatment exposures to better characterize real-world determinants of survival in this rare lymphoma subtype.

## 2. Materials and Methods

### 2.1. Study Design and Data Source

This retrospective cohort study used data from the National Cancer Institute’s SEER program to evaluate sociodemographic factors and treatment patterns associated with overall survival among patients with SMZL [15]. The study period spanned 2000 through 2022. SEER is a population-based registry that captures incident cancers, selected initial treatment indicators, vital status, and cause of death for defined geographic regions in the United States. The dataset includes patient-level demographic characteristics, tumor and staging variables as available, treatment recodes, follow-up time, and vital status. This study used deidentified, publicly available SEER registry data and qualifies as non-human subject research. Accordingly, institutional review board/ethics committee review and approval were not required in accordance with U.S. federal regulations governing research using publicly available deidentified data.

### 2.2. Inclusion and Exclusion Criteria

Inclusion Criteria: Patients were eligible if they had (1) histologically confirmed splenic marginal zone lymphoma defined using ICD-O-3 histology code 9689 and (2) a primary site in the spleen (ICD-O-3 site code C42.2).

**Figure 1:**
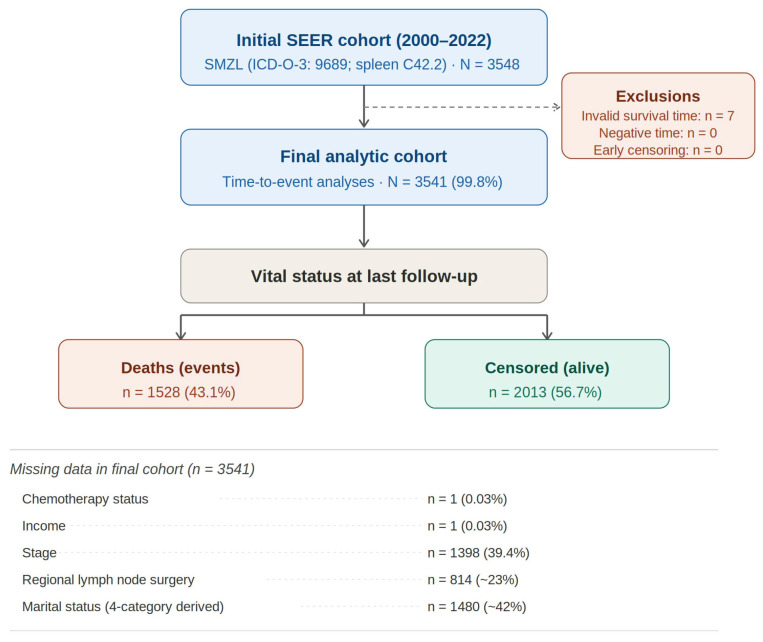
Study selection flowchart.

Exclusion Criteria: Patients were excluded if required variables for time-to-event analyses were missing or invalid (e.g., missing survival time or event indicator) or if the patient was diagnosed in an autopsy. Figure 1 shows the study selection flowchart.

### 2.3. Study Variables

Sociodemographic characteristics included sex, race, Hispanic ethnicity, marital status at diagnosis, county-level rurality, and county-level median household income. Sex was analyzed as female versus male; race was grouped as White, Black, Asian/Pacific Islander, American Indian/Alaska Native, or unknown; ethnicity was classified as Hispanic or non-Hispanic; marital status was categorized as married, single, divorced, separated, widowed, unmarried/domestic partner, or unknown. Marital status was defined at diagnosis as recorded in SEER and analyzed as a baseline covariate; changes over time are not captured in the registry. Rurality was measured using a seven-level county classification, and median household income, adjusted to 2023 United States Dollars, was presented as increasing income categories. Clinical factors included age and year at diagnosis, both treated as continuous variables, and Ann Arbor stage, categorized as stages I–IV, or unknown/missing. Treatment variables included receipt of systemic antineoplastic therapy, radiation therapy, primary site surgery, and regional lymph node surgery, with treatments analyzed according to whether they were received and, for surgical variables, by the extent or type of procedure, with infrequent categories retained and unknown or not applicable categories used as reference where appropriate. Primary site surgery (splenectomy) was included as a treatment variable given its historical role in SMZL management and its evaluation in prior population-based studies, allowing assessments of real-world treatment patterns captured in SEER despite not representing a current standard first-line modality [13,17,18]. Chemotherapy was defined based on SEER treatment recode variables and includes systemic therapy as recorded in the registry; however, SEER does not reliably distinguish specific agents, and therefore, this category may include rituximab and other immunochemotherapy regimens without the ability to differentiate them. Hence, this variable was described as “systemic antineoplastic therapy” in this study. This variable also does not distinguish between “no” and “unknown”. The timing of treatment initiation is not available, precluding time-dependent modeling.

### 2.4. Outcomes

The primary endpoint was overall survival (OS), defined as the time from diagnosis to death from any cause; individuals alive at last follow-up were censored. Survival time was measured in months using the SEER survival time variable.

Disease-specific survival (DSS) was not selected as the primary endpoint because SEER cause-of-death attribution is derived from death certificate coding and may be misclassified, particularly for indolent lymphomas, where death may occur from treatment toxicity, infections, secondary malignancies, or comorbid disease rather than direct lymphoma progression. This limitation is especially relevant in SMZL, which predominantly affects older adults with substantial competing risks of non-lymphoma mortality. Accordingly, use of DSS in this context may introduce differential misclassification and bias across sociodemographic groups. In this study, the distribution of causes of death demonstrated prominent non-lymphoma mortality (e.g., cardiovascular, pulmonary, infectious, and other causes), which limits the interpretability of DSS and increases susceptibility to differential misclassification across sociodemographic strata. Therefore, overall survival was selected as the primary endpoint, as it represents a more robust and clinically meaningful measure for population-based analyses in this setting (Supplemental Table S1).

### 2.5. Statistical Analysis

Descriptive statistics were used to summarize baseline characteristics and treatment patterns. Continuous variables were reported as mean (SD), and categorical variables were reported as frequency (percentage).

Time-to-event analyses were conducted using Kaplan–Meier methods to estimate survival distributions by selected categorical predictors. Differences in survival curves were evaluated using log-rank tests (pooled over strata). Median survival times and corresponding standard errors and confidence intervals were reported when estimable; the results for categories with very small sample sizes were interpreted cautiously, given the instability of estimates.

Associations between covariates and OS were evaluated using Cox proportional hazards regression. Univariate Cox models were fit for each predictor separately. For categorical variables, indicator (dummy) coding was used as appropriate. Continuous predictors (age, year of diagnosis, and median household income ordinal category) were modeled per 1-unit increase as presented in the output.

A multivariable Cox model was then fit, including covariates meeting the prespecified inclusion threshold from univariate analyses (*p* < 0.10) and those carried forward in the reported multivariable output with indicator parameter coding. The final adjusted model included sex, year of diagnosis, race, ethnicity, marital status, regional lymph node surgery, and systemic antineoplastic therapy. Adjusted hazard ratios (HRs) with 95% CIs were reported. Two-sided *p* values of <0.05 were considered statistically significant. The proportional hazards assumption was assessed using time-dependent covariates.

For missing data, univariate Cox proportional hazards models were performed using available-case analysis. Multivariable Cox regression was conducted using complete-case analysis, including only individuals with complete data for all covariates. The rural–urban continuum indicator-coded model demonstrated non-convergence/singularity due to sparse categories, and estimates from that model were considered unstable and were not emphasized in inference. All analyses were performed using IBM SPSS Statistics (v31, IBM Corp., Armonk, NY, USA).

### 2.6. Sensitivity and Subgroup Analysis

To evaluate the robustness of the findings to missing data, a sensitivity analysis was performed by excluding variables with substantial missingness from the multivariable model.

## 3. Results

### 3.1. Cohort Characteristics

A total of 3548 patients with histologically confirmed primary splenic marginal zone lymphoma were identified in SEER (2000–2022). The mean age at diagnosis was 68.2 years (SD 11.6), and 53.6% were female. Most patients were White (89.8%) and non-Hispanic (92.1%). Marital status at diagnosis was most commonly married (57.9%), followed by widowed (14.6%), single (11.0%), divorced (9.4%), and unknown (6.3%); separated (0.5%) and unmarried/domestic partner (0.3%) were uncommon. Most patients resided in metropolitan counties (≥1 million population, 61.3%). Ann Arbor stage was recorded as stage IV in 38.9% and stage I in 13.2%; however, staging was missing in 39.4% of cases (Table 1).

With respect to initial treatment, 26.4% received systemic antineoplastic therapy, 0.7% received beam radiation, and 21.4% underwent primary site surgery. Regional lymph node surgery was uncommon: 49.9% had no lymph node surgery, 1.5% had 1–3 nodes removed, and 0.7% had ≥4 nodes removed; lymph node surgery was unknown/not applicable in 40.4% (Table 1).

### 3.2. Cause of Deaths

In total, 56.8% were alive at the last follow-up. Among decedents, the most frequent recorded cause of death was non-Hodgkin lymphoma (15.8% of the entire cohort), followed by major competing causes including diseases of the heart (6.1%) and a heterogeneous category of other causes of death (5.2%). Additional non-lymphoma causes each contributed smaller proportions, most commonly chronic respiratory disease (1.7%), cerebrovascular disease (1.3%), lung cancer (1.2%), and infection-related mortality (e.g., pneumonia/influenza 0.8%, septicemia 0.4%). Collectively, this pattern indicates that while lymphoma-related mortality constitutes a substantial share of deaths, competing cardiovascular, pulmonary, infectious, and secondary malignancy causes are prominent, consistent with the older age distribution of SMZL and underscoring the importance of survivorship-focused management alongside lymphoma-directed care (Supplementary Table S1).

### 3.3. Survival Analyses

**Figure 2:**
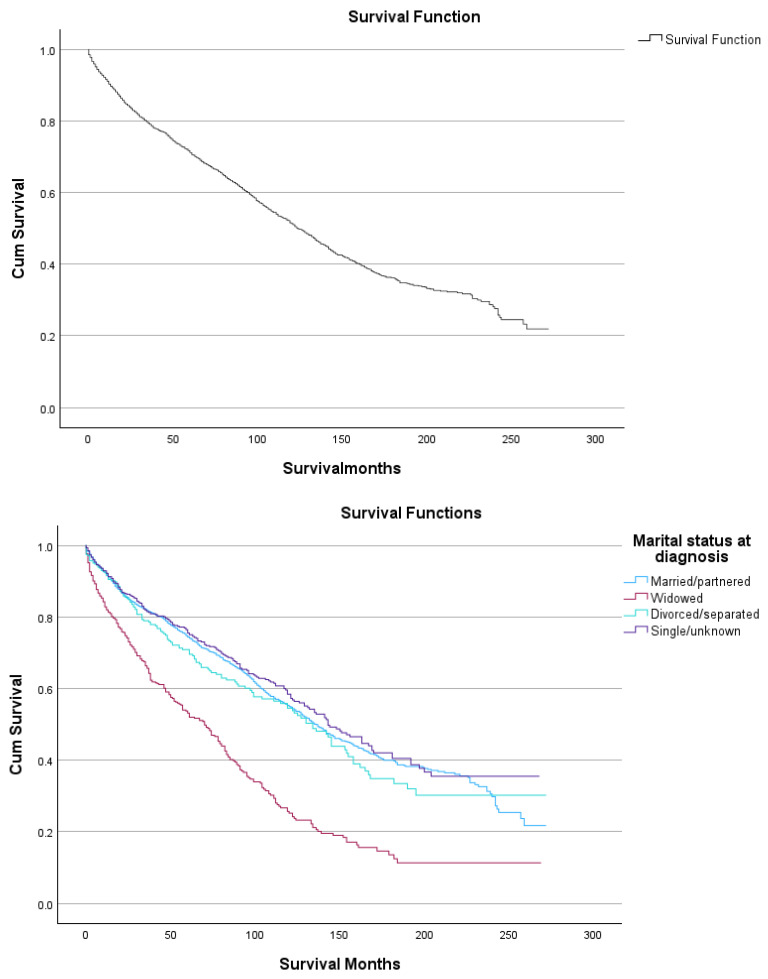

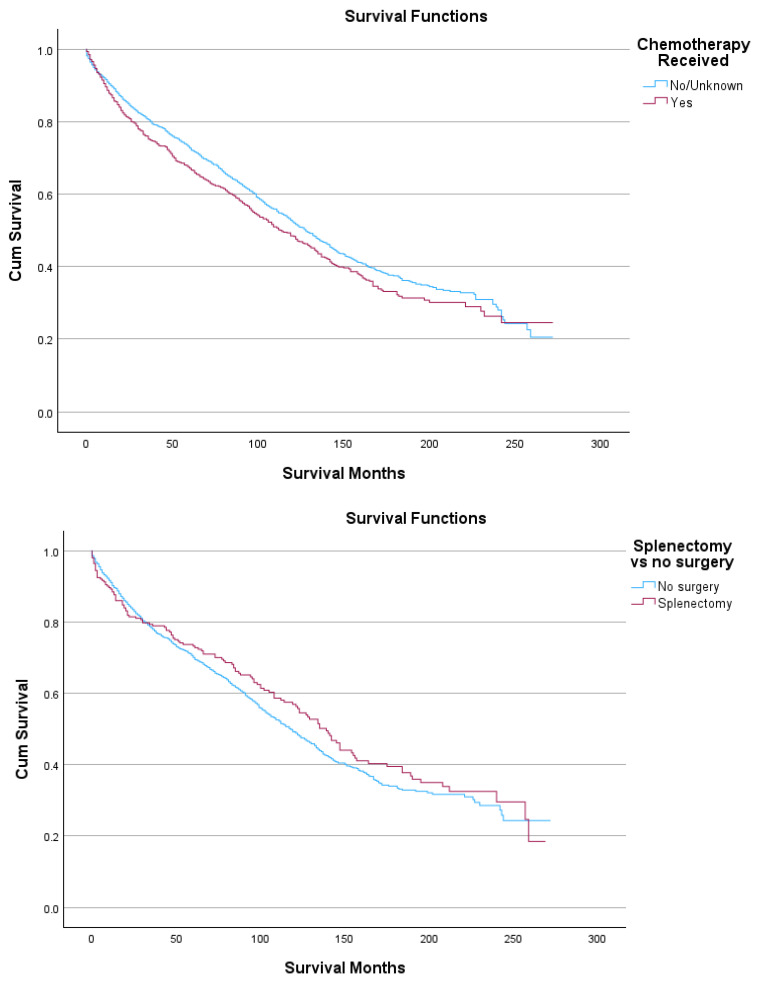
Kaplan–Meier Survival plots for overall survival in SMZL.

Kaplan–Meier analyses showed no statistically significant differences in overall survival by extent of primary site surgery (log-rank *p* = 0.376), despite numerical variations in median survival across surgical categories. Patients who underwent site-specific resection or other major surgery exhibited median survival estimates comparable to those managed without primary site surgery, while categories with very small sample sizes showed unstable estimates with wide confidence intervals, limiting interpretability. In contrast, overall survival differed markedly by marital status (log-rank *p* < 0.001), with widowed patients experiencing substantially shorter median survival (87 months) compared with married/partnered, divorced/separated, or single patients. Systemic antineoplastic therapy exposure was also associated with statistically significant differences in survival distributions (log-rank *p* = 0.013), with patients receiving no or unknown systemic antineoplastic therapy status demonstrating longer median survival (128 months) compared with those who received systemic antineoplastic therapy (113 months) (Figure 2).

### 3.4. Univariate Predictors of Overall Survival

In univariate Cox proportional hazards analyses, several demographic and clinical factors were significantly associated with overall survival. Increasing age was strongly associated with higher mortality risk (HR 1.07 per year, 95% CI 1.066–1.077; *p* < 0.001). Male sex (HR 1.17, *p* = 0.003), Hispanic ethnicity (HR 1.22, *p* = 0.030), and receipt of systemic antineoplastic therapy (HR 1.15, *p* = 0.013) were also associated with worse survival, whereas non-White race was associated with a modestly lower hazard of death (HR 0.90, *p* = 0.003). Higher income was protective (HR 0.98 per category, *p* = 0.002). Marital status demonstrated a strong association, with widowed patients experiencing significantly increased mortality compared to married individuals (HR 2.15, *p* < 0.001), while divorced and single patients did not differ significantly. Advanced-stage disease was associated with worse outcomes, particularly stage IV (HR 1.67, *p* = 0.001). Radiation therapy was associated with improved survival when comparing beam radiation to no radiation (HR 0.54, *p* = 0.006), whereas lymph node surgery, splenectomy, rural–urban status, and year of diagnosis were not significantly associated with survival (Table 2).

### 3.5. Multivariable Predictors of Overall Survival

On multivariable Cox regression, older age (HR 1.082, 95% CI 1.072–1.091; *p* < 0.001), male sex (HR 1.338, 95% CI 1.139–1.571; *p* < 0.001), Hispanic ethnicity (HR 1.422, 95% CI 1.079–1.875; *p* = 0.012), systemic antineoplastic therapy use (HR 1.416, 95% CI 1.178–1.704; *p* < 0.001), divorced/separated marital status (HR 1.353, 95% CI 1.033–1.773; *p* = 0.028), and stage IV disease (HR 1.701, 95% CI 1.159–2.496; *p* = 0.007) were associated with worse overall survival, whereas race, radiation, income, and year of diagnosis were not independently associated with survival (Table 3). The proportional hazards assumption was assessed using Schoenfeld residuals. The global test was not significant (*p* = 0.33), indicating no evidence of violation. All covariates satisfied the proportional hazards assumption, although systemic antineoplastic therapy showed borderline evidence of time-varying effects (*p* = 0.051) (Supplemental Table S2).

### 3.6. Sensitivity Analysis

In sensitivity analysis excluding variables with substantial missingness, multivariable Cox regression demonstrated consistent results. Increasing age (HR 1.072 per year, 95% CI 1.067–1.078; *p* < 0.001) and male sex (HR 1.331, 95% CI 1.198–1.479; *p* < 0.001) remained independently associated with worse overall survival. Hispanic ethnicity (HR 1.436, 95% CI 1.202–1.715; *p* < 0.001) and receipt of systemic antineoplastic therapy (HR 1.259, 95% CI 1.130–1.404; *p* < 0.001) were also associated with increased mortality. The Black race was associated with worse survival compared with White patients (HR 1.385, 95% CI 1.077–1.782; *p* = 0.011), whereas other racial groups were not significantly different. Higher income was associated with improved survival (HR 0.976 per unit, 95% CI 0.962–0.991; *p* = 0.001). Marital status remained significant overall (*p* < 0.001), with widowed (HR 1.316, *p* < 0.001) and divorced/separated patients (HR 1.394, *p* < 0.001) demonstrating worse outcomes compared with married/partnered individuals. Radiation therapy was not significantly associated with survival (*p* = 0.092). More recent year of diagnosis was associated with improved survival (HR 0.983, 95% CI 0.973–0.993; *p* = 0.001) (Table 4).

## 4. Discussion

To our knowledge, this study represents one of the largest population-based cohort of primary SMZL reported to date. In multivariable analysis, age, sex, Hispanic ethnicity, marital status, year of diagnosis, race, and systemic antineoplastic therapy exposure were independently associated with OS. Increasing age was the strongest continuous predictor of mortality. Male sex remained associated with a higher risk of death. Hispanic ethnicity was associated with inferior survival compared with non-Hispanic ethnicity. Race was not independently associated with overall survival in the adjusted model, although estimates were imprecise for smaller subgroups. These findings are consistent with prior reports in indolent B cell lymphomas that document survival differences across demographic groups [10,13,19,20].

Marital status retained a significant association with OS after adjustment. In the adjusted model, divorced/separated marital status was associated with worse survival, while widowed status showed a borderline association and single status was not significantly different. The prior oncology literature has reported survival differences by marital status [9,10,11,12,13,14,15,16,19,20,21]. Our analysis confirms that marital status remains associated with OS in SMZL within a population-based cohort.

In the context of SMZL, an indolent disease predominantly affecting older adults, the magnitude and persistence of the marital status association suggest that social determinants and supportive care factors play a central role in shaping long-term outcomes. This interpretation is reinforced by the observed distribution of causes of death, which showed a substantial contribution from non-lymphoma causes, including cardiovascular, pulmonary, infectious, and secondary malignancy-related mortality [13,22]. The observed associations between sociodemographic factors and overall survival may be influenced by residual confounding. SEER does not capture important clinical and contextual variables such as comorbidities, performance status, disease burden, access to care, or treatment selection factors, which may differ across sociodemographic groups and contribute to survival differences.

Primary site surgery and radiation therapy were not associated with differences in OS in univariate analyses. Systemic antineoplastic therapy exposure was associated with was associated with increased risk of death in the adjusted model; however, this finding likely reflects treatment-selection bias (confounding by indication), as patients receiving systemic therapy may have had more advanced or symptomatic disease. In addition, radiation estimates were unstable due to very small sample size and showed direction reversal after adjustment, consistent with confounding. These findings should be interpreted within the context of registry-based data. SEER does not capture treatment intent, specific regimens, dose intensity, performance status, or detailed measures of disease burden. As a result, the observed association between systemic antineoplastic therapy and OS most likely reflects underlying disease severity and treatment selection rather than a direct adverse treatment effect [5,6,13]. Residual confounding by disease severity is likely substantial, as SEER lacks data on performance status, tumor burden, treatment intent, and indications for therapy. However, these results provide valuable population-level insight into real-world practice patterns while underscoring the need for prospective clinical data to define treatment efficacy.

Stage IV disease was associated with worse overall survival in univariate analysis, while earlier stages were not significantly different. Although the stage was not recorded in 39.4 percent of cases, the available data reflect real-world registry reporting patterns in SMZL and allow evaluation of survival within a large, contemporary population-based cohort. The absence of a strong stage signal is consistent with prior observations that traditional anatomic staging has limited prognostic discrimination in SMZL [13,20].

Our population-based cohort is broadly consistent with prior epidemiologic descriptions of SMZL, which characterize it as a rare malignancy (≈0.13 per 100,000 person-years; ≈0.6–1% of non-Hodgkin lymphoma) affecting predominantly older adults with a median age at diagnosis of approximately 65–70 years and no consistent sex predominance [5,23]. The age distribution in our cohort (mean: 68.2 years; median: 70 years) closely aligns with these prior reports, reinforcing the established demographic profile of SMZL. Similarly, the predominance of White, non-Hispanic patients in our cohort is consistent with earlier SEER-based analyses, although the degree of racial homogeneity observed here (≈90% White) is somewhat more pronounced and may reflect both underlying population structure and registry capture patterns [5,24]. Importantly, prior population-based studies have largely focused on basic demographic variables and survival outcomes, whereas our analysis extends these observations by incorporating social and geographic variables, including marital status, rural–urban residence, and income, providing a more comprehensive characterization of real-world patient context in SMZL.

Consistent with prior reports, our cohort demonstrates a substantial proportion of patients with advanced-stage disease alongside significant missingness in staging data, reflecting the known limitations of applying Ann Arbor staging to splenic- and marrow-predominant lymphomas in registry datasets [24,25]. Notably, while earlier population-based analyses have described SMZL as an indolent lymphoma with favorable survival (5-year relative survival ≈80%) without robust stage-stratified modeling, our findings provide more granular evidence that advanced stage (stage IV) is independently associated with worse overall survival [5,23]. With respect to treatment patterns, our results reflect a shift from historical paradigms: Whereas earlier series emphasized splenectomy as a dominant first-line strategy, only approximately one-fifth of patients in our cohort underwent primary site surgery, while systemic therapy was administered in roughly one-quarter, underscoring increasing heterogeneity in real-world management [25,26]. In line with prior population-based studies, we did not observe a significant survival advantage associated with splenectomy, supporting the view that surgical management primarily serves symptom control rather than long-term survival benefit [24]. The observed association between systemic therapy and worse overall survival contrasts with outcomes reported in clinical and institutional series but is most plausibly explained by confounding by indication and residual bias, as patients receiving therapy in routine practice are more likely to have advanced or symptomatic disease, a pattern well described in non-randomized registry analyses of indolent lymphomas [27].

Our survival findings are also consistent with the established indolent natural history of SMZL, with more than half of patients alive at last follow-up and a relatively modest proportion of deaths attributable directly to lymphoma, paralleling prior SEER-based estimates of favorable long-term outcomes [23,24]. However, our study adds important granularity by demonstrating the substantial contribution of non-lymphoma causes of death, including cardiovascular, pulmonary, infectious, and secondary malignancy-related mortality, thereby reinforcing the central role of competing risks in this older population [24]. Beyond traditional clinical prognostic factors, our multivariable analysis confirms the importance of age and stage while extending prior models by demonstrating independent associations of sex, ethnicity, marital status, and socioeconomic context with survival. In particular, the adverse impact of male sex and Hispanic ethnicity, along with the protective effect of higher income, aligns with the broader lymphoma disparity literature but has been insufficiently characterized in SMZL-specific cohorts [26]. Notably, the strong and persistent association between marital status and survival, particularly worse outcomes among widowed and divorced/separated patients, highlights the influence of social support and care context, representing a novel population-level signal that extends beyond traditional biologic prognostic models and underscores the dual contribution of disease biology and social determinants in shaping outcomes in SMZL [5,24].

The overall survival patterns observed in this study should be interpreted within the context of evolving treatment paradigms in SMZL. The treatment landscape for indolent B-cell lymphomas, including SMZL, has evolved with the introduction of targeted agents such as Bruton tyrosine kinase inhibitors (e.g., zanubrutinib) [28]. These therapies have demonstrated high response rates and favorable tolerability in relapsed or refractory marginal zone lymphoma and may further improve disease control and long-term outcomes [29]. As a result, survival estimates derived from this cohort may not fully reflect outcomes achievable with contemporary therapies. As uptake of these agents increases in clinical practice, future population-based analyses incorporating treatment-specific data will be important to better define their effect on survival outcomes in SMZL.

The extended study period (2000–2022) spans substantial changes in SMZL management, including a shift from splenectomy-based approaches to immunotherapy and the more recent introduction of targeted agents [1,4,30]. These evolving treatment paradigms may influence survival patterns over time, and outcomes observed in earlier years of the cohort may not fully reflect those achievable with contemporary therapies.

This study has several notable strengths, including a large population-based cohort, extended follow-up, and broad geographic representation. These features provide robust and generalizable estimates of survival in a rare lymphoma subtype that is not feasible to study in randomized trials. Although registry data do not capture detailed clinical variables or treatment intent, including chemotherapy regimen details, comorbidities, performance status, and disease burden, they offer a valuable real-world perspective on outcomes across diverse patient populations. Despite inherent limitations such as stage missingness and limited treatment granularity, the consistency and magnitude of the observed associations support the validity of the findings. The high proportion of missing stage data (~40%) may introduce bias and limits interpretation of stage-adjusted models, particularly if missingness is not random. Within this framework, our results demonstrate that demographic and social variables remain independently associated with OS in SMZL. These data provide clinically relevant insight and reinforce the importance of comprehensive assessment, survivorship planning, and comorbidity management in this predominantly older patient population.

## 5. Conclusions

In this large population-based analysis of SMZL, overall survival was independently associated with age, sex, ethnicity, marital status, and systemic antineoplastic therapy exposure. Male sex, Hispanic ethnicity, advancing age, and receipt of systemic antineoplastic therapy were associated with was associated with increased risk of death, while married status was associated with improved survival. Local treatment approaches, including splenectomy and radiation, were not associated with differences in survival. The substantial contribution of non-lymphoma causes of death highlights the importance of competing health risks in this older population. These findings represent associations from registry-based observational data and should be interpreted accordingly.

## Figures and Tables

**Table 1:** Baseline demographic, clinical, and treatment characteristics (*N* = 3548).

Characteristic	*n* (%) or Mean ± SD/Median (IQR)
**Age at diagnosis, years**	68.21 ± 11.60/70 (17)
**Sex**	
Female	1900 (53.6)
Male	1648 (46.4)
**Race**	
White	3186 (89.8)
Black	164 (4.6)
Asian/Pacific Islander	155 (4.4)
American Indian/Alaska Native	13 (0.4)
Unknown	30 (0.8)
**Ethnicity**	
Non-Hispanic	3268 (92.1)
Hispanic	280 (7.9)
**Marital status at diagnosis**	
Married	2055 (57.9)
Widowed	519 (14.6)
Single (never married)	391 (11.0)
Divorced	333 (9.4)
Separated	18 (0.5)
Unmarried/domestic partner	10 (0.3)
Unknown	222 (6.3)
**Rural–urban residence**	
Metro ≥ 1,000,000	2175 (61.3)
Metro 250,000–1,000,000	700 (19.7)
Metro < 250,000	227 (6.4)
Nonmetro adjacent to metro	242 (6.8)
Nonmetro not adjacent	201 (5.7)
Unknown/missing	3 (0.1)
**Median household income (inflation-adjusted to 2023)**	
<$40,000	31 (0.9)
$40,000–$44,999	42 (1.2)
$45,000–$49,999	62 (1.7)
$50,000–$54,999	104 (2.9)
$55,000–$59,999	124 (3.5)
$60,000–$64,999	226 (6.4)
$65,000–$69,999	210 (5.9)
$70,000–$74,999	273 (7.7)
$75,000–$79,999	470 (13.2)
$80,000–$84,999	366 (10.3)
$85,000–$89,999	407 (11.5)
$90,000–$94,999	188 (5.3)
$95,000–$99,999	207 (5.8)
$100,000–$109,999	350 (9.9)
$110,000–$119,999	215 (6.1)
$120,000+	272 (7.7)
Unknown/missing	1 (0.0)
**Ann Arbor stage**	
Stage I	470 (13.2)
Stage II	127 (3.6)
Stage III	73 (2.1)
Stage IV	1381 (38.9)
Unknown/Missing	1497 (42.2)
**Treatment**	
Systemic antineoplastic therapy (yes)	938 (26.4)
Systemic antineoplastic therapy (no/unknown)	2610 (73.6)
Beam radiation	25 (0.7)
Radiation none/unknown	3521 (99.2)
Radiation refused	2 (0.1)
Primary site surgery (spleen)	761 (21.4)
**Regional lymph node surgery**	
None	1769 (49.9)
1–3 nodes removed	54 (1.5)
≥4 nodes removed	25 (0.7)
Biopsy/aspiration	5 (0.1)
Nodes removed unknown number	32 (0.9)
Blank/Unknown	1663 (46.9)

**Table 2:** Univariate Cox proportional hazards models for overall survival.

Variable	B	SE	HR (Exp(B))	95% CI	Wald	*p*-Value
Sex (Male vs. Female)	0.153	0.051	1.17	1.05–1.29	8.93	0.003
Race (Non-White vs. White)	−0.105	0.036	0.90	0.84–0.97	8.61	0.003
Hispanic Ethnicity	0.195	0.090	1.22	1.02–1.45	4.73	0.030
Systemic Antineoplastic Therapy (Yes vs. No)	0.136	0.055	1.15	1.03–1.28	6.17	0.013
Radiation (Beam vs. None)	−0.615	0.225	0.54	0.35–0.84	7.47	0.006
Radiation (Refused vs. None)	0.375	1.025	1.46	0.20–10.85	0.13	0.715
Year of Diagnosis	−0.009	0.005	0.99	0.98–1.00	3.10	0.078
Income (Ordinal)	−0.023	0.007	0.98	0.96–0.99	9.64	0.002
Marital status (Widowed vs. Married)	0.765	0.067	2.15	1.89–2.45	130.93	<0.001
Divorced vs. Married	0.109	0.090	1.12	0.94–1.33	1.47	0.225
Single vs. Married	−0.064	0.075	0.94	0.81–1.09	0.73	0.395
Age (Continuous)	0.069	0.003	1.07	1.07–1.08	693.42	<0.001
LN Surgery (Yes vs. No)	−0.007	0.161	0.99	0.73–1.36	0.00	0.966
Stage II vs. I	0.159	0.096	1.17	0.97–1.42	2.72	0.099
Stage III vs. I	0.180	0.137	1.20	0.92–1.57	1.72	0.190
Stage IV vs. I	0.515	0.159	1.67	1.23–2.29	10.48	0.001
RUCC (Metro < 1M vs. ≥1M)	0.098	0.060	1.10	0.98–1.24	2.70	0.100
Non-Metro vs. ≥1M	0.121	0.077	1.13	0.97–1.31	2.46	0.117
Splenectomy (Yes vs. No)	−0.106	0.093	0.90	0.75–1.08	1.30	0.255

HR = Hazard ratio (Exp(B)). Reference categories: female, White, non-Hispanic, no systemic antineoplastic therapy, no radiation, married, stage I, metro ≥ 1M, no surgery. Continuous variables: age, income, and year of diagnosis. For categorical variables with >2 levels, global Wald test is significant where noted (e.g., marital status, stage).

**Table 3:** Multivariable Cox proportional hazards model for overall survival.

Variable	B	SE	Wald	*p*-Value	HR (95% CI)
**Age (Per Year)**	0.079	0.005	298.71	<0.001	1.08 (1.07–1.09)
**Sex**					
Male vs. Female	0.291	0.082	12.52	<0.001	1.34 (1.14–1.57)
**Race (Ref: White)**			4.16	0.385	
American Indian/Alaska Native	−0.669	1.003	0.45	0.505	0.51 (0.07–3.66)
Asian/Pacific Islander	−0.133	0.265	0.25	0.614	0.88 (0.52–1.47)
Black	0.261	0.216	1.46	0.227	1.30 (0.85–1.98)
Unknown	−1.397	1.002	1.95	0.163	0.25 (0.04–1.76)
**Hispanic Ethnicity**					
Hispanic vs. Non-Hispanic	0.352	0.141	6.24	0.012	1.42 (1.08–1.88)
**Systemic Antineoplastic Therapy**					
Yes vs. No/Unknown	0.348	0.094	13.66	<0.001	1.42 (1.18–1.70)
**Radiation**					
Beam vs. None/Unknown	0.467	0.362	1.67	0.197	1.60 (0.79–3.25)
**Income (Ordinal)**	−0.007	0.011	0.37	0.544	0.99 (0.97–1.02)
**Marital status (Ref: Married)**			7.13	0.068	
Widowed	0.210	0.109	3.74	0.053	1.23 (1.00–1.53)
Divorced/Separated	0.303	0.138	4.83	0.028	1.35 (1.03–1.77)
Single/Unknown	0.071	0.119	0.35	0.553	1.07 (0.85–1.36)
**Stage (Ref: Stage I)**			12.51	0.006	
Stage II	−0.010	0.149	0.00	0.947	0.99 (0.74–1.33)
Stage III	0.139	0.182	0.59	0.444	1.15 (0.81–1.64)
Stage IV	0.531	0.196	7.35	0.007	1.70 (1.16–2.50)
**Year of Diagnosis**	−0.018	0.012	2.06	0.152	0.98 (0.96–1.01)

Abbreviations: HR, hazard ratio; CI, confidence interval; SE, standard error. Multivariable Cox proportional hazards regression was performed using complete-case analysis, including only patients with non-missing data for all covariates (*n* = 2066). Reference categories were as follows: female (sex), White (race), non-Hispanic (ethnicity), no/unknown systemic antineoplastic therapy, no/unknown radiation, married/partnered (marital status), and stage I disease. The proportional hazards assumption was assumed for all variables.

**Table 4:** Sensitivity analysis: multivariable Cox proportional hazards model.

Variable	B	SE	Wald	*p*-Value	HR (95% CI)
**Age (Per Year)**	0.070	0.003	630.689	<0.001	1.072 (1.067–1.078)
**Male vs. Female**	0.286	0.054	28.327	<0.001	1.331 (1.198–1.479)
**Race (Overall)**	—	—	13.565	0.009	—
American Indian/Alaska Native vs. White	−0.829	0.503	2.720	0.099	0.436 (0.163–1.169)
Asian/Pacific Islander vs. White	−0.004	0.140	0.001	0.975	0.996 (0.757–1.310)
Black vs. White	0.326	0.128	6.441	0.011	1.385 (1.077–1.782)
Unknown Race vs. White	−1.451	0.708	4.198	0.040	0.234 (0.058–0.939)
**Hispanic vs. Non-Hispanic**	0.362	0.091	15.945	<0.001	1.436 (1.202–1.715)
**Systemic Antineoplastic Therapy: Yes vs. No/Unknown**	0.231	0.056	17.256	<0.001	1.259 (1.130–1.404)
**Radiation: Beam vs. None/Unknown**	0.383	0.227	2.846	0.092	1.467 (0.940–2.289)
**Income (Ordinal, Per Unit)**	−0.024	0.007	10.367	0.001	0.976 (0.962–0.991)
**Marital Status (Overall)**	—	—	22.923	<0.001	—
Widowed vs. Married/Partnered	0.275	0.073	14.134	<0.001	1.316 (1.141–1.519)
Divorced/Separated vs. Married/Partnered	0.332	0.092	13.162	<0.001	1.394 (1.165–1.667)
Single/Unknown vs. Married/Partnered	0.116	0.076	2.347	0.126	1.123 (0.968–1.304)
**Year of Diagnosis (Per Unit)**	−0.017	0.005	10.609	0.001	0.983 (0.973–0.993)

HR, Hazard ratio; CI, confidence interval. Cox proportional hazards regression performed using available-case analysis; event defined as death (dead = 1); time to event defined as survival months; *N* = 3541 included in analysis (1528 events, 2013 censored); reference categories were female, White race, non-Hispanic ethnicity, no/unknown systemic antineoplastic therapy, no/unknown radiation, and married/partnered. Hazard ratios reported with 95% confidence intervals; *p*-values derived from Wald tests; categorical variables modeled using indicator (dummy) coding; continuous variables (age, income, year of diagnosis) modeled per unit increase.

## Data Availability

The data used in this study are publicly available through the Surveillance, Epidemiology, and End Results Program (SEER), National Cancer Institute. Access to SEER data requires completion of a data use agreement and can be obtained at https://seer.cancer.gov.

## Supplementary Materials

The following supporting information can be downloaded at https://www.mdpi.com/article/10.3390/cancers18081300/s1. Table S1: Cause-of-death distribution (COD to site recode) in the SMZL cohort (SEER 2000–2022; *N* = 3548); Table S2: Assessment of proportional hazards assumption using Schoenfeld residuals.
