# Supplementary material for: Sociodemographic Factors and Treatment Patterns Associated with Overall Survival in Splenic Marginal Zone Lymphoma: A Nationwide Retrospective Cohort Study (2000–2022)
Source: Cancers (Basel). 2026 Apr 20;18(8):1300. doi: 10.3390/cancers18081300 (PMC13114261; doi:10.3390/cancers18081300)
Supplement: Supplementary file 1 [file cancers-18-01300-s001.zip › cancers-4187282-supplementary.pdf]

Supplemental Table S1. Cause-of-death distribution (COD to site recode) in the SMZL cohort (SEER 2000–2022; *N* = 3548).

Cause of death (COD to site recode)	*n*	%
Alive	2015	56.8
Non-Hodgkin Lymphoma	562	15.8
Diseases of Heart	217	6.1
Other Cause of Death	184	5.2
Chronic Obstructive Pulmonary Disease and Allied Cond	61	1.7
Cerebrovascular Diseases	46	1.3
Lung and Bronchus	43	1.2
Miscellaneous Malignant Cancer	32	0.9
Pneumonia and Influenza	28	0.8
Accidents and Adverse Effects	26	0.7
Chronic Lymphocytic Leukemia	23	0.6
Diabetes Mellitus	23	0.6
Alzheimers (ICD-9 and 10 only)	21	0.6
State DC not available or state DC available but no COD	20	0.6
Hypertension without Heart Disease	17	0.5
Other Infectious and Parasitic Diseases including HIV	17	0.5
Pancreas	16	0.5
Acute Myeloid Leukemia	13	0.4
Colon excluding Rectum	13	0.4
Aleukemic, Subleukemic and NOS	12	0.3
In situ, benign or unknown behavior neoplasm	12	0.3
Nephritis, Nephrotic Syndrome and Nephrosis	12	0.3
Symptoms, Signs and Ill-Defined Conditions	11	0.3
Other Lymphocytic Leukemia	9	0.3
Melanoma of the Skin	9	0.3
Prostate	9	0.3
Breast	7	0.2

Chronic Liver Disease and Cirrhosis	6	0.2
Liver	6	0.2
Stomach	5	0.1
Biopsy/aspiration of regional lymph node, NOS	5	0.1
Hodgkin Lymphoma	4	0.1
Esophagus	4	0.1
Suicide and Self-Inflicted Injury	4	0.1
Aortic Aneurysm and Dissection	3	0.1
Atherosclerosis	3	0.1
Congenital Anomalies	3	0.1
Myeloma	3	0.1
Other Acute Leukemia	3	0.1
Ovary	3	0.1
Brain and Other Nervous System	2	0.1
Acute Lymphocytic Leukemia	2	0.1
Chronic Myeloid Leukemia	2	0.1
Other Myeloid/Monocytic Leukemia	2	0.1
Other Diseases of Arteries, Arterioles, Capillaries	2	0.1
Tongue	2	0.1
Gallbladder	1	0.0
Anus, Anal Canal and Anorectum	1	0.0
Corpus Uteri	1	0.0
Intrahepatic Bile Duct	1	0.0
Kidney and Renal Pelvis	1	0.0
Larynx	1	0.0
Non-Melanoma Skin	1	0.0
Other Biliary	1	0.0
Other Urinary Organs	1	0.0
Rectum and Rectosigmoid Junction	1	0.0
Salivary Gland	1	0.0

Soft Tissue including Heart	2	0.1
Septicemia	14	0.4
Ureter	1	0.0
Urinary Bladder	3	0.1
**Total**	**3548**	**100.0**

Supplementary Table S2. Assessment of proportional hazards assumption using Schoenfeld residuals.

Variable	ϱ (rho)	χ^2	df	p-value
Age	0.0236	0.42	1	0.517
Sex (Male vs Female)	-0.0161	0.17	1	0.683
Race (overall)	—	—	4	0.336
American Indian/Alaska Native vs White	-0.0381	0.93	1	0.336
Asian or Pacific Islander vs White	-0.0448	1.27	1	0.260
Black vs White	-0.0489	1.54	1	0.215
Hispanic (vs Non-Hispanic)	-0.0161	0.17	1	0.684
Systemic antineoplastic therapy (Yes vs No/Unknown)	-0.0767	3.81	1	0.051
Radiation (Beam vs None/Unknown)	0.0130	0.11	1	0.741
Income (ordinal)	-0.0570	2.20	1	0.138
Marital status (overall)	—	—	3	0.933
Widowed vs Married/partnered	0.0033	0.01	1	0.933
Divorced/separated vs Married/partnered	-0.0122	0.10	1	0.757
Single/unknown vs Married/partnered	-0.0125	0.10	1	0.752
Stage (overall)	—	—	3	0.748
Stage II vs Stage I	-0.0129	0.10	1	0.748
Stage III vs Stage I	-0.0041	0.01	1	0.917
Stage IV vs Stage I	-0.0646	2.94	1	0.086
Year of diagnosis	-0.0146	0.14	1	0.712
**Global test**	—	17.84	16	0.333
